# Analysis of health related quality of life (HRQoL) of patients with clinically localized prostate cancer, one year after treatment with external beam radiotherapy (EBRT) alone versus EBRT and high dose rate brachytherapy (HDRBT)

**DOI:** 10.1186/1748-717X-3-20

**Published:** 2008-07-15

**Authors:** Kurian Jones Joseph, Riaz Alvi, David Skarsgard, Jon Tonita, Nadeem Pervez, Cormac Small, Patricia Tai

**Affiliations:** 1 Department of Radiation Oncology, Cross Cancer Institute & University of Alberta, Edmonton, Alberta, Canada; 2 Department of Epidemiology Saskatchewan Cancer Agency, Regina, Saskatchewan, Canada; 3 Department of Radiation Oncology Tom Baker Cancer Center & University of Calgary, Calgary, Alberta, Canada; 4 Department of Radiation Oncology, Allan Blair Cancer Centre and University of Saskatchewan, Regina, Saskatchewan, Canada

## Abstract

**Purpose:**

Prostate cancer is the leading form of cancer diagnosed among North American men. Most patients present with localized disease, which can be effectively treated with a variety of different modalities. These are associated with widely different acute and late effects, which can be both physical and psychological in nature. HRQoL concerns are therefore important for these patients for selecting between the different treatment options.

**Materials and methods:**

One year after receiving radiotherapy for localised prostate cancer 117 patients with localized prostate cancer were invited to participate in a quality of life (QoL) self reported survey. 111 patients consented and participated in the survey, one year after completion of their treatment. 88 patients received EBRT and 23 received EBRT and HDRBT. QoL was compared in the two groups by using a modified version of Functional Assessment of Cancer Therapy-Prostate (FACT-P) survey instrument.

**Results:**

One year after completion of treatment, there was no significant difference in overall QoL scores between the two groups of patients. For each component of the modified FACT-P survey, i.e. physical, social/family, emotional, and functional well-being; there were no statistically significant differences in the mean scores between the two groups.

**Conclusion:**

In prostate cancer patients treated with EBRT alone versus combined EBRT and HDRBT, there was no significant difference in the QoL scores at one year post-treatment.

## Background

Prostate cancer is the leading form of cancer diagnosed among Canadian men and accounts for approximately 27% of new cancer cases and 11.4% of cancer deaths [1,2]. Most newly diagnosed patients with prostate cancer present with localized disease [3]. EBRT is a treatment option for patients with early-stage or locally advanced prostate cancer [4]. Recent data suggest that local control of prostate cancer is directly related to the radiation dose which would in turn result in improved biochemical control, disease-free survival and increased overall survival [5]. Various dose escalation methods are available including three-dimensional conformal radiotherapy (3D-CRT) or intensity-modulated radiotherapy (IMRT) with or without low-dose rate (LDR), high-dose rate (HDR) or particle beam boost [6-9].

Health related quality of life (HRQoL) is a subjective measure of a patient's perception of well-being. HRQoL concerns are important for patients with prostate cancer for selecting between different treatments options which may result in physical and psychological sequelae that would affect their daily lives. Evaluation of the quality of life (QoL) among cancer patients who had received radiation using different radiotherapeutic techniques certainly helps both patients and physicians to make a more informed decision on their treatment.

The main objective of this study was to compare prospectively HRQoL at one year after treatment in prostate cancer patients treated with either EBRT alone or a combination of EBRT and iridium HDRBT.

## Patients and methods

### Patient characteristics

This is a prospective study carried out among patients with localized prostate cancer during the period January 2000 to December 2002, in the department of Radiation Oncology at the Allan Blair Cancer Center (ABCC), in Regina, Canada. One of the standard treatment options for these patients during this period was EBRT alone. We started the prostate HDRBT program at the ABCC in 1999. Since then, HDRBT was used as an alternative method of dose escalation in patients with locally advanced prostate cancer who have received EBRT.

Patients in our study (Table 1) belonged to the intermediate or high risk groups with the following features: histological diagnosis of adenocarcinomas of the prostate, pretreatment PSA ≤ 20 ng/ml (mean), clinical stage T1c -T3a, prostate volume ≤ 60 cc, no evidence of lymphadenopathy on the pelvic CT scan, and a negative bone scan. During the above time period, 117 patients met the above criteria were treated at our institution with either EBRT alone or a combination of EBRT plus HDRBT.

**Table 1:** Characteristic of the study population

	EBRT group	RBRT+HDRB group
No of patients	88	23
Median age (Range)	69(46–84)	69(50–78)
Tumor stage		
T1	20	4
T2	59	15
T3	9	4
Gleason score		
2–6	55	14
7	21	7
8–10	12	2
Pretreatment PSA (ng/ml)	Mean: 14.1	Mean: 12.3
	Median: 8.7	Median: 8.9
	Range: 1.7 – 161.8	Range: 0.8 – 51.9
≤ 10	46	14
10–20	31	5
≥ 20	11	4
Radiotherapy dose	66–70 Gy/33–35 Fr	40 Gy+16.5 Gy Brachy

### Radiotherapy details

All the patients who received EBRT were treated by using 3D-CRT with a 4-field box technique using 10-MV or higher-energy photons. The EBRT alone group received a total dose of 66–70 Gy in 33 – 35 fractions (2-Gy per fraction) over six and half to seven weeks. This was the standard EBRT dose during that period (although considered to be low compared to current standards). EBRT was delivered in two phases. The planning target volume (PTV) for the initial phase included the prostate gland and seminal vesicles with a margin of 1–2 cm receiving a dose of 40 Gy in 20 fractions (2-Gy per fraction) over four weeks. The boost phase PTV for EBRT only group was defined as the prostate with a margin of 0.7–1 cm and received a dose of 26–30 Gy in 13–15 fractions (2-Gy per fraction) over two and half to three weeks at the discretion of the treating Radiation Oncologist. None of the patients received regional nodal radiation. Patients who were in the EBRT plus HDRBT treatment group received HDRBT initially followed by EBRT. Iridium-192 HDRBT was performed via a transperineal approach, delivering a total dose of 16.5 Gy in 3 fractions within 24 hours with a minimum gap of six hours between fractions. PTV for the brachytherapy treatment consists of the entire prostate gland and capsule with a margin of 2–3 mm. EBRT was given two weeks after HDRBT delivering a dose of 40 Gy at 2 Gy per fraction over 4 weeks.

### Quality of life questionnaire

All one hundred and seventeen patients who underwent either EBRT alone or a combination of EBRT plus HDRBT were invited to participate in an institutional review board approved self-reported QoL survey at one year after completion of their radiation treatment. One hundred and eleven patients consented and participated in the survey. Eighty-eight patients of 111 received EBRT alone and 23 patients received combined EBRT and HDRBT. The survey was conducted using a modified version of the Functional Assessment of Cancer Therapy-Prostate (FACT-P) survey instrument (version 3). Results between the two treatment groups were compared using a paired t-test.

## Results

Characteristics of the study population, according to treatment received, are shown in Table 1. Median age was identical between patients treated with EBRT alone and those treated with EBRT and HDRB. Approximately 2/3 of patients in both groups had stage T2 disease. There were no significant differences between the two groups with respect to the distribution of established risk factors (stage, Gleason score and PSA).

Table 2 shows modified FACT-P scores at one year for the two different treatment groups. There was no significant difference in overall QoL between the two groups (p = 0.668). For each component of the modified FACT-P survey, i.e. Physical Well-Being, Social/Family Well-Being, Emotional Well-Being, and Functional Well-Being, there was no statistically significant difference in the mean scores between the two groups.

**Table 2:** Comparison of Modified FACT-P scores of two treatment groups.

HRQoL Component:	Mean score		p-value
		
	EBRT + HDRBT	EBRT	
Physical Well -Being	6.86	6.59	0.865
Social/Family Well-Being:	19.26	17.96	0.289
Emotional Well-Being:	9.76	9.99	0.865
Functional Well-Being:	28.78	27.13	0.252
Prostate cancer subscale:	16.86	18.38	0.447
Overall:	97.64	95.04	0.668

## Discussion

Patients with prostate cancer can live for many years regardless of the treatment they receive due to the long natural history. As a result, HRQoL has become an important outcome measure in patients with this disease. Different survey instruments are designed to assess HRQoL of patients with prostate cancer [3,10,11]. We have conducted our survey by using a modified version of FACT-P (version 3) survey tool [12,13] The structure of FACT-P (version 3) survey instrument comprising a 47-item questionnaire, which is divided into four primary QoL domains: physical, social/family, emotional, and functional well-being, plus a 12-item prostate cancer subscale. These 12-items ask about symptoms and problems specific to prostate cancer. Higher total scores for the FACT-P scale indicate a better overall QoL. The modified survey instrument has a total of 50 questions and the only modification is in the prostate subscale for the purpose of accommodating symptoms related to radiation induced late toxicity (Table 3).

**Table 3:** Modified FACT-P Prostate subscale (version3). Questions 47–49 added to the FACT-P Prostate subscale (version3)

Additional Concern		Not at all	A little bit	Some- what	Quite a bit	Very much					
35.	I am losing weight.......................................	0	1	2	3	4					
36.	I have a good appetite...................................	0	1	2	3	4					
37.	I have aches and pains that bother me	0	1	2	3	4					
38.	I have certain areas of my body where I experience significant pain	0	1	2	3	4					
39.	My pain keeps me from doing things I want to do...	0	1	2	3	4					
40.	I am satisfied with my present comfort level..........	0	1	2	3	4					
41.	I am able to feel like a man..............................	0	1	2	3	4					
42.	I have trouble moving my bowels.....................	0	1	2	3	4					
43.	I have difficulty urinating.......................................	0	1	2	3	4					
44.	I urinate more frequently than usual...................	0	1	2	3	4					
44.	I have difficulty starting to urinate......................	0	1	2	3	4					
45.	My problems with urinating limit my activities......	0	1	2	3	4					
46	I am able to have and keep an erection	0	1	2	3	4					
47.	I have rectal bleeding....................................	0	1	2	3	4					
48.	I have diarrhea.............................................	0	1	2	3	4					
49.	I have blood in my urine.................................	0	1	2	3	4					
50.	Looking at the above 15 questions, how much would you say these ADDITIONAL CONCERNS affect your quality of life? (circle one number)										
	0	1	2	3	4	5	6	7	8	9	10
	Not at all									Very much so	

Sathya *et al *reported a randomized study in which patients with locally advanced prostate cancer were treated with EBRT alone or combination of EBRT and iridium HDRBT. The study showed no difference in the toxicity scores between the two arms at 18 months of follow up [9]. This study also provided evidence that higher doses of radiation delivered by the combination treatment resulted in better local as well as biochemical control in locally advanced prostate cancer. Other studies have also reported improved local control following dose escalation with 3D-CRT or IMRT [7,8]. The risks of long-term morbidity, following dose escalation by various methods are incompletely understood yet and they could have a significant impact on post treatment QoL.

Various studies have demonstrated the significance of HRQoL assessment when considering different treatment options for prostate cancer and suggested that recommended treatment decisions should take into account HRQoL in addition to survival [12,13]. Wei *et al *reported a comparative HRQoL outcome study for patients with localized prostate cancer who underwent brachytherapy, radical prostatectomy or EBRT [14]. Higher FACT-P scores were reported in patients treated with EBRT than with brachytherapy. The authors concluded that the HRQoL changes are likely to be treatment-specific. Welsh *et al *compared the baseline to six months post treatment QoL of 10 patients with prostate cancer treated with EBRT plus HDRBT [15]. They found that the median QoL scores were comparable to baseline values at six months and concluded that EBRT plus HDRBT is an acceptable treatment when QoL is considered.

Our study compared HRQoL among patients with localized prostate cancer who had undergone radical treatment using the two different radiation techniques. Our findings indicated that both groups had similar QoL outcomes at the end of one year of treatment. Vordermark D *et al *reported the results of a similar study among 84 prostate cancer patients treated with either EBRT alone or EBRT plus HDRBT [16]. The study showed comparable QoL data between the two groups at a median duration of 19 months post treatment. HRQoL differences may not become apparent at the end of one to two years of completion of treatment and hence a longer follow up might be helpful to see the difference. Wahlgren T *et al *reported the five year disease-specific HRQoL of patients with localized prostate cancer following combined treatment including EBRT, HDRBT and hormone therapy [17]. The long term data reported that only minor differences in general HRQoL compared with normative data. We are in the process of reviewing HRQoL of our patients at five years post treatment.

Our data may have significant implications. While offering curative radiation treatment, most of the patients are interested to know how the different treatments compare to one another in terms of survival, long term morbidity and HRQoL. Our study showed the significance of the evaluation of QoL which would help both patients and physicians to make more informed decisions between different treatment techniques.

A potential limitation of our study is that this is a single-institutional non-randomised study with a small sample size in the EBRT plus HDRBT arm. In addition the study lacks baseline HRQoL information. Radiation doses are also low by today's standards. However the study does help to minimise concerns of using combination treatment as an alternative for dose escalation when QoL is considered.

## Conclusion

Our study showed that the impact of prostate cancer treatments such as EBRT alone vs. combination of EBRT and HDRBT on HRQoL was comparable at one year after completion of treatment. Prospective randomized studies are needed to confirm these findings. In addition, an understanding of the relative HRQoL would help clinicians and patients to make informed choices between different treatment options that may offer a similar chance of tumor control.

## Competing interests

The authors declare that they have no competing interests.

## Authors' contributions

KJJ wrote the manuscript and supported with data analysis. RA collected the data, enrolled patients and performed the statistical analysis. DS critically reviewed the manuscript. JT critically reviewed the manuscript. NP critically reviewed the manuscript. CS critically reviewed the manuscript. PT supported the data collection, enrolled patients and critically reviewed the manuscript. All authors read and approved the final manuscript.
